# Serum IL-1, Pyroptosis and Intracranial Aneurysm Wall Enhancement: Analysis Integrating Radiology, Serum Cytokines and Histology

**DOI:** 10.3389/fcvm.2022.818789

**Published:** 2022-01-27

**Authors:** Qingyuan Liu, Yisen Zhang, Chengcheng Zhu, Weiqi Liu, Xuesheng Ma, Jingang Chen, Shaohua Mo, Linggen Dong, Nuochuan Wang, Jun Wu, Peng Liu, Hongwei He, Shuo Wang

**Affiliations:** ^1^Department of Neurosurgery, Beijing Tiantan Hospital, Capital Medical University, Beijing, China; ^2^China National Clinical Research Center for Neurological Diseases, Beijing, China; ^3^Department of Neurointevention, Beijing Tiantan Hospital, Capital Medical University, Beijing, China; ^4^Neurosurgical Institution, Beijing Tiantan Hospital, Capital Medical University, Beijing, China; ^5^Department of Radiology, University of Washington, Seattle, WA, United States; ^6^Medical Image Center, Tongxinyilian (Unimed), Tsinghua Tongfang Science and Technology Mansion, Beijing, China; ^7^Department of Blood Transfusion, Beijing Tiantan Hospital, Capital Medical University, Beijing, China

**Keywords:** unruptured intracranial aneurysm, aneurysm wall enhancement, pyroptosis, serum cytokines, IL-1

## Abstract

**Background and Purpose:**

Aneurysm wall enhancement (AWE) is correlated with the rupture and growth risk of unruptured intracranial aneurysms (UIAs). Pyroptosis is a proinflammation mode of lytic cell death, mediated by pyroptosis-related proteins, i.e., gasdermin D and interleukin 1 β (IL-1β). Integrating serum cytokines and histology, this study aimed to investigate the correlation between AWE and pyroptosis in UIAs.

**Methods:**

UIA patients receiving microsurgical clipping were prospectively enrolled from January 2017 and June 2020. UIA samples were collected, as well as the corresponding blood samples. In this study, high-resolution magnetic resonance was employed to identify the AWE. The serum 46-cytokines examination and the histological analysis were conducted to determine pyroptosis, CD68 and MMP2. The IL-1 ra/beta ratio was determined by complying with the serum IL-1β and IL-1.ra. A comparison was drawn in the differences between UIAs with and without AWE. Lastly, the correlation between inflammation in UIA samples and serums was investigated.

**Results:**

This study included 34 UIA patients. The serum proinflammatory cytokines [IL-1β (*P* < 0.001) and TNF-α (*P* < 0.001)] were up-regulated, and serum anti-inflammatory cytokine (IL-1.ra, *P* = 0.042) were down-regulated in patients with AWE UIAs. The patients with AWE UIAs achieved a higher IL-1.ra/beta ratio (*P* < 0.001). The multivariate logistic analysis demonstrated IL-1β [odds ratio (OR), 1.15; 95% confidence interval (CI), 1.02–1.30; *P* = 0.028] and IL-1.ra (OR, 0.998; 95% CI, 0.997–1.000; *P* = 0.017) as the risk factors correlated with the AWE. IL-1.ra/beta ratio achieved the highest predictive accuracy [area under the curve (AUC), 0.96] for AWE, followed by IL-1.ra (AUC, 0.90), IL-1β (AUC, 0.88) and TNF-α (AUC, 0.85). As compared with the UIAs without AWE, the AWE UIAs were manifested as a severer wall remodeling, with higher relative levels of pyroptosis-related proteins, CD68 and MMP2. The serum IL-1β, IL-1.ra and IL-1.ra/beta ratio had a positive correlation with the relative levels of pyroptosis-related proteins, CD68 and MMP2 in UIA tissues.

**Conclusion:**

The serum IL-1β and IL-1.ra were correlated with the AWE. More pyroptosis-related proteins were identified in UIAs with AWE. The serum IL-1β and IL-1.ra were correlated with the pyroptosis-related proteins in aneurysm tissues.

## Introduction

Intracranial aneurysm is the major cause of non-traumatic subarachnoid hemorrhage. The rupture or growth risk of unruptured intracranial aneurysms (UIAs) is critical to clinical decision-making. Aneurysm wall enhancement (AWE) is considered a sign of rupture, growth and symptoms (1–5). Inflammation infiltration and aneurysm wall remodeling are recognized as two vital pathological characteristics of the wall of ruptured intracranial aneurysm (6). As revealed from existing studies, the inflammation is severer in the aneurysm wall of UIAs with AWE as compared with UIAs without AWE (7, 8). As revealed by the fact above, the pathological features of UIAs with AWE may be close to those of the ruptured intracranial aneurysms. Though strong AWE may indicate a high risk of aneurysm rupture or growth, the mechanism of AWE and the correlation between AWE and the characteristics of systematic inflammation have not been clarified.

Pyroptosis is a proinflammation mode of lytic cell death, which is mediated by Gasdermin D (GSDMD) and interleukin 1β (IL-1β) (9). Caspase-1 is capable of cleaving pro-IL-1β to the bioactive cytokine and cleaving the pro-GSDMD to GSDMD products. In addition, the GSDMD products can create pores to mediate IL-1β efflux and mitigate the subsequent inflammation (10). Pyroptosis is of high significance to numerous acute and chronic inflammation diseases (11, 12). Since the UIAs with AWE are generally manifested as massive inflammation infiltration, a hypothesis has been proposed that pyroptosis may be related to AWE.

This study conducted the analysis integrating serum cytokines and the histological analysis, which aimed to clarify the correlation between AWE, pyroptosis in aneurysm tissues and systematic inflammation conditions.

## Methods

### Study Population

We prospectively enrolled UIA patients between January 2017 and June 2020. The inclusion criteria included: (1) saccular and single UIA; (2) the aneurysm size >5 mm (the size of UIA sufficiently large for resection after clipping); (3) vessel wall magnetic resonance imaging scans performed preoperatively to detect the AWE of UIAs; (4) UIAs were treated by microsurgical clipping. The exclusion criteria are presented below: (1) patients with other intracranial tumors (e.g., pituitary adenoma) and cerebrovascular malformation (e.g., arteriovenous malformation); (2) patients with dissecting, traumatic, thrombotic or infectious UIAs; (3) patients with a family history of UIAs, polycystic kidney or systematic disease (e.g., lupus); (4) patients receiving special treatment in other medical institutions before the admission to the authors' institution. Lastly, 34 patients harboring 34 UIAs were included in this study.

The demographic information (e.g., age, gender, smoking history and alcohol abuse history) comorbidities [e.g., hypertension, dyslipidemia, diabetes mellitus and transient ischemic attacks (TIA)] and medication (aspirin and antihyperlipidemic drugs) were acquired from electronic medical records.

We defined patients taking aspirin (e.g., standard and low-dose aspirin) at least three times per week as aspirin users (13). The patients treated with standard antihyperlipidemic drugs were considered the antihyperlipidemic drug users. Based on the alcohol consumption, the included patients were classified as regular alcohol abuse (drink once or more per week) and others (14). Based on the smoking condition, patients were classified into current smokers and others (15).

### Radiological Characteristics

Computed tomography angiography (CTA) was routinely performed before the surgery. The aneurysm size, height, diameter of neck and diameter of the parent artery were determined by complying with CTA by two independent investigators (YSZ and QYL, who work as vascular neurosurgeons for over 5 year, and were blind to clinical information). The mentioned characteristics were measured twice by the respective investigator, and the average of the respective investigator was then averaged for the in-depth analysis. Subsequently, the aspect ratio (AR) and the size ratio (SR) were determined following the previous study by the authors (16). AR denotes the ratio of aneurysm size and aneurysm neck diameter, and SR represents the ratio of aneurysm height and diameter of the parent artery.

**Figure 1:**
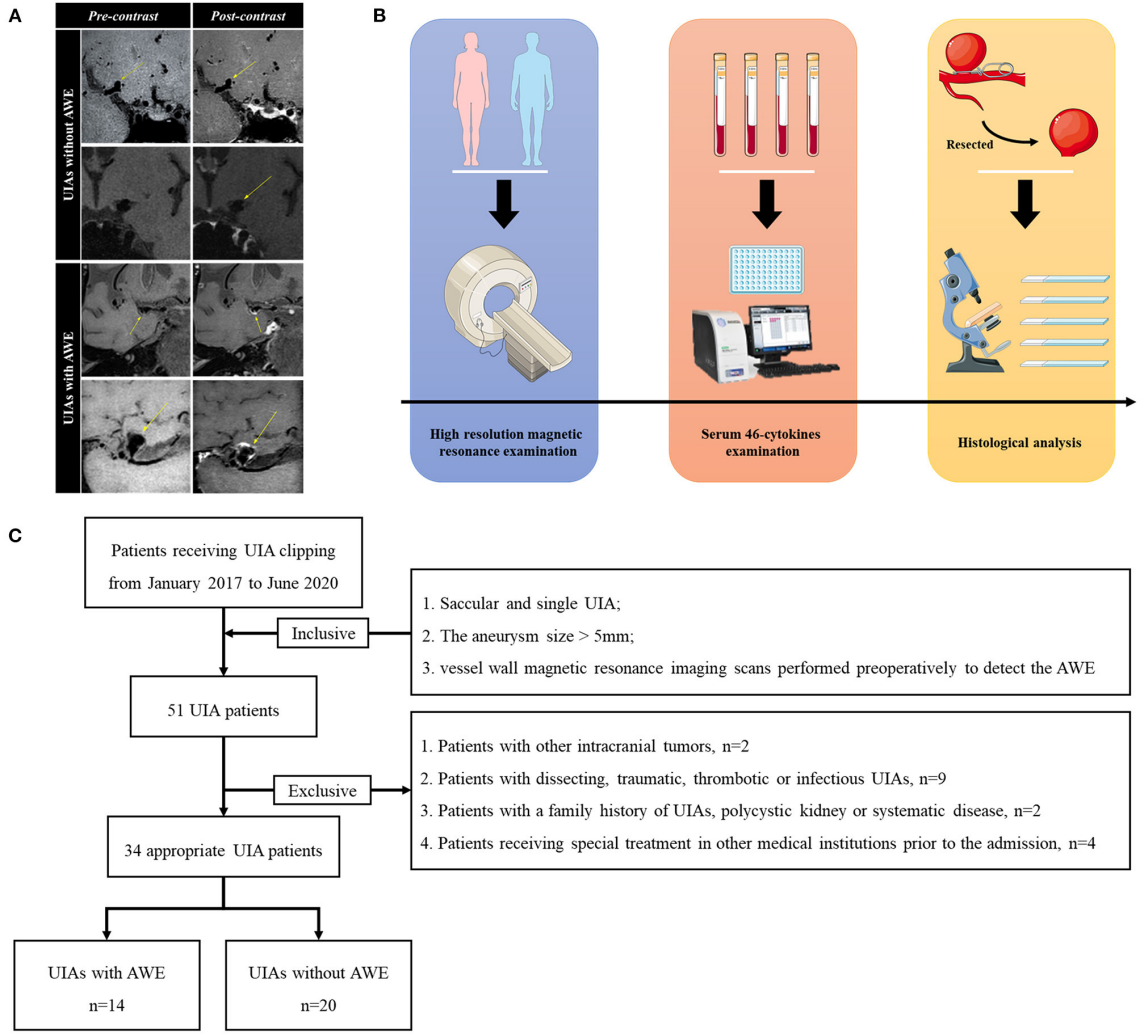
The diagram of analysis. **(A)** Four representative cases of UIAs with and without AWE. To be specific, the pre-contrast and post-contrast statues of UIAs were presented. **(B)** The diagram of analysis. Preoperatively, the appropriate UIA patients would receive high-resolution magnetic resonance examinations. The blood samples of the mentioned patients would be tested with the 46-cytokines examination. After appropriate clipping, the UIAs would be resected for the histological analysis. **(C)** The flow chart of patient enrollment. This study finally recruited 34 UIA patients. UIA, unruptured intracranial aneurysm; AWE, aneurysm wall enhancement.

The same investigators (YSZ and QYL) recorded the UIAs with and without AWE based on vessel wall magnetic resonance imaging scans. The routine sequences of vessel wall magnetic resonance imaging scans consisted of pre-contrast and post-contrast T1-weighted, as well as time-of-flight (TOF). All images were acquired using the same magnetic resonance image workstation (Siemens, Germany). TOF was used to locate the UIAs with a repetition time (TR)/echo time (TE) of 3.43/21 ms, a layer thickness of 0.60 mm, a field of view of 220 × 200 mm, 52 slices, resolution of 384 × 256 under a scan time of 6 min 8 s. Vessel wall sequence was used to identify the AWE with TR/TE of 900/20 ms, a layer thickness of 0.65 mm, a field of view of 230 × 195 mm, 240 slices, resolution of 384 × 384 under a scan time of 8 min 4 s. By referencing the existing study (3), the investigators identified the UIAs with AWE when the aneurysm wall was presented to be partial (the partial aneurysm wall enhanced) and circumferential (the whole aneurysm wall enhanced) AWE by comparing the pre-contrast images with the post-contrast images. Figure 1A presents the representative cases.

### Serum Cytokines Detection and Histological Analysis

Figure 1B gives the diagram of analysis. After the vessel wall magnetic resonance imaging scans, the blood sample was acquired from the respective patient for the serum cytokines examination preoperatively. The cytokines were detected through a serum 46-cytokines examination (Wayenbio corporation, Shanghai, China) using Bio-Plex Human Cytokines Screening Panel (Bio-Rad corporation, Hercules, American). Serum samples were centrifuged for 10 min at 15,000 rpm. The supernate was collected and diluted using the standard diluent. Then, Microbeans were diluted using the Assay Buffer and incubated with samples for 30 min. Finally, each sample was mixed with Detection Antibody and then detected by Bio-Plex MAGPIX system (Bio-Rad corporation, Hercules, American). The function of each cytokine was summarized in Supplementary Table 1. The fold change (FC) of UIAs with AWE to those without AWE was determined. The cytokines with FC >2 and *P* < 0.05 were defined as the significantly different cytokines. The IL-1 ra/beta ratio was calculated by:


$$\text{IL}-1\text{ ra}/\text{beta ratio}=\frac{\left(\text{serum IL}-1.\text{ra}\right)}{\left(\text{serum IL}-1\text{β}\right)}$$

The result of serum 46-cytokines examination was further validated using the Ella workstation (Biotechne corporation, Shanghai, China). The Simple Plexes were purchased from the Biotechne corporation, which was incubated using the primary antibodies, including IL-1β, interleukin 1 ra (IL-1.ra), tumor necrosis factor-α (TNF-α) and interleukin 10 (IL-10) (R&D Systems, Biotechne corporation, Shanghai, China).

The protein-to-protein interaction (PPI) analysis was performed based on STRNG database (https://www.string-db.org/).

Subsequently, the dome of UIAs was resected after the appropriate clipping for the histological analysis. After the dehydration and the embedding, the sections were first stained with H-E (Hematoxylin-Eosin) and Masson (Solabrio, Beijing, China). In addition, the EVG (Verhoeff Van Gieson) staining was performed by Raisedragon's corporation (Beijing, China). For immunofluorescence, after antigen retrieval and permeabilization, the sections were blocked with ProteinBlock (Abcam corporation, Cambridge, UK) and then incubated with primary antibodies at 4°C overnight, including rabbit anti-CD68 (ab125212, Abcam corporation, Cambridge, UK) at 1:500, mouse anti-MMP2 antibody (ab86607, Abcam corporation, Cambridge, UK) at 1:500, rabbit anti-IL-1β antibody (ab9722, Abcam corporation, Cambridge, UK) at 1:300, Alexa Fluor® 488 Anti-IL-1.ra antibody (ab252007, Abcam corporation, Cambridge, UK) at 1:500, as well as rabbit anti-GSDMD antibody (ab219800, Abcam corporation, Cambridge, UK) at 1:500. Subsequently, the sections were incubated with secondary antibodies (Alexa Fluor® 488 Goat anti-mouse IgG, Abcam corporation, Cambridge, UK, and Alexa Fluor® 594 Goat anti-rabbit IgG, Abcam corporation, Cambridge, UK) at 1:500. Lastly, the sections were sealed with the DAPI (4',6-diamidino-2-phenylindole)-containing mounting medium (ZSGB-BIO corporation, Beijing, China). In this study, the sections with identical conditions and set integration times were scanned under the microscopy workstation (Invitrogen™ EVOS™ FL Auto 2, Thermofisher scientific corporation, Massachusetts, USA). All fluorescence channels of the whole sample were automatically recorded for further analysis. The Image J 1.8.0 (https://imagej.en.softonic.com/) was employed to measure the integrated optical density (IOD) and count the cell number by complying with the DAPI image. As for cell number count, we firstly transferred the DAPI image into 8-bit image, and then adjusted the threshold of Black and White model to select cells. After the holes filling and watershed processes, the cell number was semi-automatically analyzed using the Analyze Particles model. The ratio of IOD to cells was used for the subsequent analysis.

Based on the H-E staining and by referencing previous studies (17, 18), three random sections were classified for aneurysm wall types by two investigators (JW and PL) blinded to clinical information. To be specific, the sections were (1) type I, described as endothelialized wall with organized smooth muscle cells, (2) type II&III, defined as a thickened wall with disorganized smooth muscle cells or hypocellular wall with intimal hyperplasia or luminal thrombosis, as well as (3) type IV, i.e., an extremely thin thrombosis-lined hypocellular wall. The discrepancies were solved by consulting a senior author (SW). The severest remodeling type in three random sections was taken for further analysis. We defined the type II&III and type IV aneurysm wall as AWR wall (17). The atherosclerosis area was measured based on the Masson staining according to our previous study (17). The atherosclerotic area was stained red, and the non-atherosclerotic area was stained blue. Atherosclerosis area was defined as the ratio of red area to blue area + red area.

### Statistical Analysis

Statistical analyses were conducted by applying SPSS 22.0 (IBM corporation, American) and GraphPad Prism 8 (GraphPad Software, American). The measured data were expressed as number and percentage [*n* (%)], and the continuous data were manifested as median and inter-quartile range [m (IQR)]. The inter-observer agreement between two investigators in assessing the morphological parameters (irregular shape, aneurysm size, AR and SR) was evaluated with Cohen's kappa and intraclass correlation coefficients; values of 0.60–0.80 and >0.80 were, respectively, considered to be of substantial and excellent inter-observer agreement. The logistic model was conducted to investigate the risk factors for the AWE. The parameters with statistical significance in univariate analysis were then input into multivariate logistic analysis. The multivariate logistic analysis was performed using a backward model. The result included odds ratio (OR) and 95% confidence interval (CI) The receiver operator characteristic curve was plotted, and the area under the curve (AUC) was generated to examine the predictive accuracy of parameters to AWE. The correlation analysis was conducted with Pearson's method. The coefficient (R) was adopted to assess the strength of correlation. The correlation with *R* > 0.50 and *P* < 0.05 was recognized as a strong correlation. A two-tailed *P*-value < 0.05 was assumed with statistical significance.

## Results

### The Characteristics of Patients and UIAs

On the whole, 34 UIA patients were included here (Figure 1C). Table 1 lists the characteristics of patients and UIAs. Of 34 included patients, 13 were male and 21 were female, with age ranging from 25 to 70. AWE was identified in 14 (41.2%) UIAs. 5 (35.7%) patients with AWE and 10 (50.0%) patients without AWE suffered from hypertension. 23 (67.7%) UIAs were sited in the middle cerebral artery, 5 (14.7%) were in the internal carotid artery, and 6 (17.6%) were in other arteries. The irregular rate of UIAs with AWE exceeded that of UIAs without AWE (50.0 vs. 30.0%). Inter-observer agreement between two investigators in the assessment of the morphological parameters was good (Supplementary Table 2). Furthermore, the difference was not identified in age, gender, dyslipidemia, diabetes mellitus, coronary heart diseases, TIA/ischemic stroke, aspirin taking, antihyperlipidemic drugs taking, smoking history, alcohol abuse, site, aneurysm size, irregular shape, AR and SR (all *P* > 0.05).

**Table 1:** The clinical and radiological information of patients and UIAs.

**Characteristics**	**With AWE *n* = 14**	**Without AWE *n* = 20**	***P* value**
Age-m (IQR)-years	52 (46–67)	59 (50–63)	0.592
Male-no. (%)	7 (50.0%)	6 (30.0%)	0.341
**Comorbidities-no. (%)**			
Hypertension	5 (35.7%)	10 (50.0%)	0.500
Dyslipidemia	1 (7.1%)	0 (0.0%)	0.743
Diabetes mellitus	1 (7.1%)	1 (5.0%)	0.931
Coronary heart diseases	4 (28.6%)	1 (5.0%)	0.259
TIA/ischemic stroke	2 (14.3%)	1 (5.0%)	0.666
Current smoking-no. (%)	4 (28.6%)	5 (25.0%)	0.877
Regular alcohol abuse-no. (%)	1 (7.1%)	1 (5.0%)	0.931
**Medication using-no. (%)**			
Aspirin	1 (7.1%)	2 (10.0%)	0.904
Antihyperlipidemics	2 (14.3%)	5 (25.0%)	0.616
Site-no. (%)			0.743
MCA	10 (71.4%)	13 (65.0%)	
ICA	2 (14.3%)	3 (15.0%)	
AcomA/ACA/posterior	2 (14.3%)	4 (20.0%)	
Irregular shape-no. (%)	7 (50.0%)	6 (30.0%)	0.341
Aneurysm size-m (IQR)-mm	6.8 (5.4–8.1)	6.0 (5.0–7.2)	0.416
AR-m (IQR)	1.4 (1.3–2.1)	1.2 (0.9–1.5)	0.053
SR-m (IQR)	2.1 (1.2–2.5)	1.9 (1.5–2.6)	0.796

*UIA, unruptured intracranial aneurysm; AWE, aneurysm wall enhancement; TIA, transient ischemic attack; MCA, middle cerebral artery; ICA, internal carotid artery; AcomA, anterior communicating artery; ACA, anterior cerebral artery; AR, aspect ratio; SR, size ratio*.

### Serum Cytokines Between UIAs With and Without AWE

**Figure 2:**
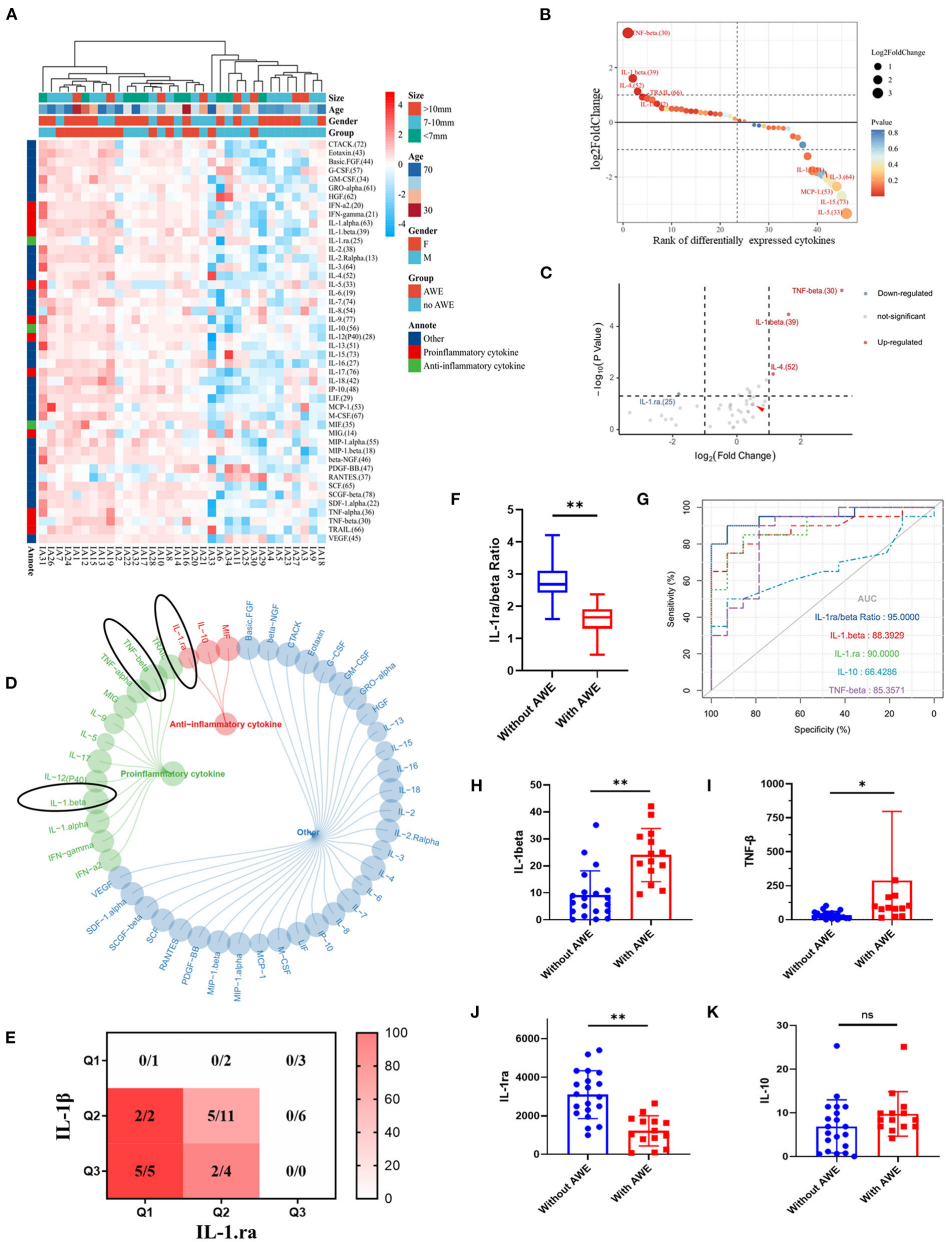
The serum cytokines between UIAs with and without AWE. **(A)** The heatmap of serum cytokines between UIAs with and without AWE. Forty-six serum cytokines were identified in this study. **(B)** The proinflammatory cytokines were up-regulated in patients with AWE UIAs, whereas the anti-inflammatory cytokines were up-regulated in patients without AWE UIAs. **(C)** The volcano plot of the different cytokines in respect of UIAs with and without AWE. FC >2 and *P*-value < 0.05 were adopted to identify the significantly different cytokines. IL-1β, TNF-α and IL-4 were signnificantly up-regulated, whereas IL-1.ra was significantly down-regulated, in patients with AWE UIAs. **(D)** The summary of the function of significantly different cytokines. The black circles indicated the cytokines of interest. **(E)** The heatmap of the AWE percentage in the respective IL-1β and IL-1.ra level. The levels of IL-1β and IL-1.ra were separated using the interquartile range. **(F)** The histograms of IL-1.ra/beta ratio for UIAs with and without AWE. **(G)** The receiver operating characteristic curves of IL-1β, IL-1.ra, IL-10, TNF-α and IL-1.ra/beta ratio to AWE. **(H–K)** The histograms of IL-1β, IL-1.ra, TNF-α and IL-10 in respect of UIAs with and without AWE. ^ns^*P* > 0.05; *P < 0.05; **P < 0.01. UIA, unruptured intracranial aneurysm; AWE, aneurysm wall enhancement; FC, fold change; IL-1β, interleukin 1β; IL-1.ra, interleukin 1.ra, TNF-α, tumor necrosis factor-α; IL-10, interleukin 10.

Forty-six cytokines between UIAs with and without AWE were found (Figure 2A; Supplementary Table 3). The proinflammatory cytokines were up-regulated progressively, whereas anti-inflammatory cytokines were down-regulated gradually in patients with AWE UIAs (Figure 2B). Four cytokines, i.e., IL-1β (*P* < 0.001), IL-1.ra (*P* = 0.042), IL-4 (*P* = 0.007) and TNF-α (*P* < 0.001), were significantly different between UIAs with AWE and without AWE (Figure 2C). Of the mentioned cytokines, IL-1β and TNF-α were proinflammatory, and IL-1.ra was anti-inflammatory (Figure 2D). Figure 2E presents the percentage of AWE in each IL-1β and IL-1.ra level. IL-1β and IL-1.ra were integrated, and a new biomarker was promoted, termed IL-1.ra/beta ratio. The UIAs without AWE had a higher IL-1.ra/beta ratio as compared with UIAs with AWE (Figure 2F). The predictive accuracy of serum cytokines to AWE was tested. As indicated by the result, IL-1.ra/beta ratio had the highest accuracy (AUC, 0.96), followed by IL-1.ra (AUC, 0.90), IL-1β (AUC, 0.88) and TNF-α (AUC, 0.85); however, an appropriate predictive accuracy of IL-10 to AWE was not identified (AUC, 0.66) (Figure 2G; Supplementary Table 4). For the IL-1β, IL-1.ra and TNF-α participated in the inflammation-related process, we mainly focused on these cytokines in the subsequent analysis.

The levels of IL-1β, IL-1.ra and TNF-α were further measured using the Ella workstation. As reported previously, IL-10, an anti-inflammatory factor, was significantly down-regulated in patients with AWE UIAs (19). IL-1β, IL-1.ra and TNF-α were significantly different between UIAs with and without AWE (Figures 2H,J), whereas IL-10 had no statistical significance (Figure 2K). Table 2 lists the result of rgw logistic analysis. As indicated by the univariate logistic analysis, IL-1β, IL-1.ra, TNF-α and IL-4 were risk factors for the AWE. The multivariate logistic analysis demonstrated IL-1β (OR, 1.15; 95% CI, 1.02–1.30; *P* = 0.028) and IL-1.ra (OR, 0.998; 95% CI, 0.997–1.000; *P* = 0.017) as the independent risk factors correlated with the AWE. The results remained consistent after being regulated. IL-1β and IL-1.ra were serum markers of pyroptosis; thus, it was assumed that AWE may be correlated with the pyroptosis in the aneurysm wall.

**Table 2:** The logistic analysis for factors related to the AWE.

**Parameters**	**Univariate**		**Multivariate** ^**†**^		**Adjusted** ^**‡**^	
	**OR (95% CI)**	***P*-value**	**OR (95% CI)**	***P*-value**	**OR (95% CI)**	***P*-value**
IL-1.β	1.17 (1.06–1.29)	0.002	1.15 (1.02–1.30)	0.028	1.16 (1.00–1.33)	0.046
IL-1.ra	0.998 (0.997–0.999)	0.004	0.998 (0.997–1.000)	0.017	0.999 (0.997–1.000)	0.038
IL-4	2.06 (1.11–3.82)	0.022	Omitted		Omitted	
TNF-α	1.03 (1.01–1.06)	0.010	Omitted		Omitted	

† *the multivariate analysis was performed using forward logistic model*.

‡ *the result was adjusted by age, gender, hypertension, dyslipidemia, diabetes mellitus, aspirin, antihyperlipidemics and aneurysm size. AWE, aneurysm wall enhancement; OR, odds ratio; CI, confidence interval*.

### Pyroptosis and Inflammation in the Aneurysm Wall Between UIAs With and Without AWE

**Figure 3:**
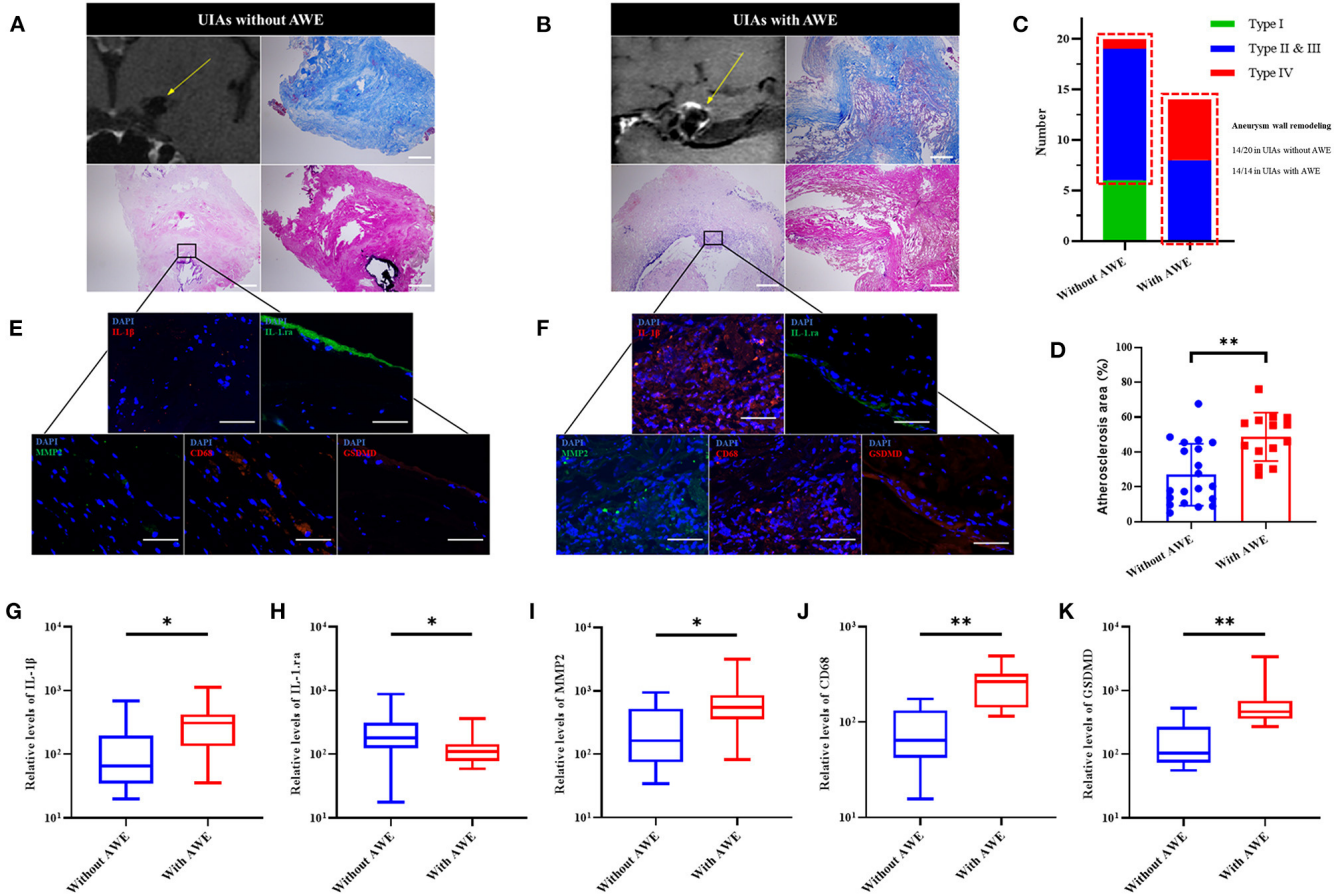
The GSDMD and the inflammatory factors in UIAs with and without AWE. **(A,B)** Two representative cases of histological analysis for UIAs with and without AWE. The H-E staining indicated a severe wall remodeling and inflammation infiltration in the UIAs with AWE. The Masson staining showed that atherosclerosis was more significantly changed in the UIAs with AWE. The EVG staining reported the reduced elastic fibers in the UIAs with and without AWE. The scale bar corresponded to 100 μm. **(C)** The histograms of aneurysm wall remodeling between UIAs with and without AWE. Thus, it was known that all UIAs with AWE were manifested as significant wall remodeling (type II & III, or type IV). The red box indicated the number of aneurysm wall remodeling. **(D)** The atherosclerosis area between UIAs with and without AWE. The UIAs with AWE had a larger atherosclerosis area compared with UIAs without AWE. **(E,F)** The IL-1β, IL-1.ra, GSDMD, CD68 and MMP2 in UIA tissues were detected in this study. The scale bar corresponding to 40 μm. **(G–K)** The box plot of the relative levels of IL-1β, IL-1.ra, MMP2, CD68 and GSDMD for UIAs with and without AWE. *P < 0.05; **P < 0.01. UIA, unruptured intracranial aneurysm; AWE, aneurysm wall enhancement; GSDMD, gasdermin D; MMP2, matrix metalloproteinase-2; IL-1β, interleukin 1β; IL-1.ra, interleukin 1.ra.

To examine the correlation more specifically between the AWE and pyroptosis in the aneurysm wall, we performed histological analysis on UIA tissue samples. Here, as compared with UIAs without AWE, a higher aneurysm wall remodeling ratio (14/20 in UIAs without AWE, and 14/14 in UIAs with AWE), severer inflammation (CD68 and MMP2) and larger atherosclerosis area could be found in UIAs with AWE (Figures 3A–D). The EVG staining detected reduced elastic fibers in UIAs with and without AWE, whereas there was no significant difference. The pyroptosis-related proteins (IL-1β, IL-1.ra and GSDMD) and the inflammatory factors were further detected in UIAs. As indicated from the result, MMP2 and CD68 were both higher in UIAs with AWE. furthermore, the UIAs with AWE had a higher relative level of IL-1β and GSDMD, and a lower relative level of IL-1.ra, as compared with UIAs without AWE (Figures 3E–K).

### The Correlation of Inflammatory Factors in UIA Tissues and Serum Cytokines

**Figure 4:**
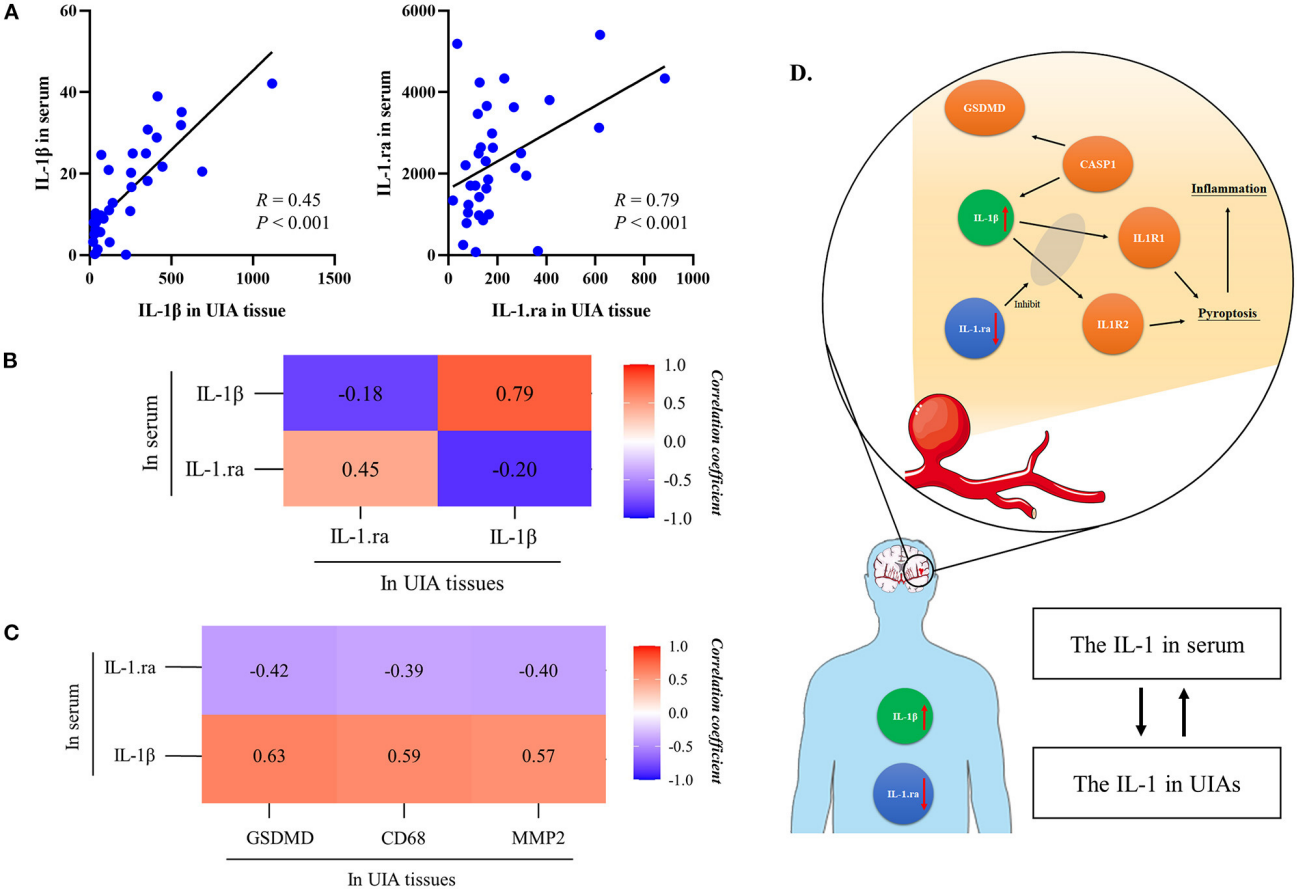
The relationship between inflammatory factors in tissue samples and serum cytokines. **(A)** The relationship of IL-1β and IL-1.ra between serum and UIA tissues. **(B)** The heatmap shows the correlation of IL-1β and IL-1.ra between serum and UIA tissues. **(C)** The heatmap presents the correlation of serum IL-1β/IL-1.ra and CD68, MMP2 in UIA tissues. **(D)** There may be a correlation between systematic proinflammatory cytokines and proinflammatory factors in UIA tissues. The IL-1β can activate the IL-1 receptor, and induce pyroptosis and subsequent inflammation. IL-1.ra can block the combination of IL-1β and IL-1 receptors. This biological process may be the mechanism of AWE, which could result in a massive inflammation infiltration. Therefore, we assumed that factors correlated with pyroptosis, e.g., IL-1.ra/beta ratio, may be a potential biomarker to predict aneurysm rupture or growth. UIA, unruptured intracranial aneurysm; GSDMD, gasdermin D; IL-1β, interleukin 1β; IL-1.ra, interleukin 1.ra.

According to Figures 4A,B, the relative level of serum IL-1β was positively correlated with the relative level of IL-1β in UIA tissues (*R* = 0.79, *P* < 0.001), the same as the serum IL-1.ra (*R* = 0.45, *P* < 0.001) and IL-1.ra/beta ratio. Moreover, according to Figure 4C, the serum IL-1β, IL-1.ra and IL-1.ra/beta ratio were correlated with the relative levels of GSDMD, CD68 and MMP2 in UIA tissues. However, the correlation of IL-4 or TNF-α and the inflammatory factors (CD68 and MMP2) in UIA tissues were not significant. The result of correlation analysis was summarized in Supplementary Table 5. As revealed from the protein-to-protein interaction analysis based on STRING database (Supplementary Figure 1), GSDMD can interact with the IL-1β, and with the IL-1.ra indirectly; the serum IL-1β can activate the IL-1 receptor, and induce pyroptosis and subsequent inflammation; IL-1.ra can inhibit the combination of IL-1β and IL-1 receptors. Thus, the biological efficacy of IL-1β and IL-1.ra is antagonistic.

## Discussion

Pyroptosis is critical to numerous inflammation diseases. This study first investigated the correlation between AWE and pyroptosis. Integrating the serum cytokines and the histological analysis, this study found that the proinflammatory cytokines (IL-1β, IL-4 and TNF-α) were up-regulated, and the anti-inflammatory cytokine (IL-1.ra) was down-regulated, in patients with UIAs showing AWE. The IL-1.ra/beta ratio had the highest predictive accuracy for AWE. The subsequent analysis showed that serum IL-1β and IL-1.ra were correlated with the relative levels of pyroptosis-related proteins and the inflammatory factors in UIA tissues. The pyroptosis-related proteins, e.g., IL-1β, IL-1.ra and IL-1.ra/beta ratio, may serve as a potential biomarker to predict the aneurysm rupture or growth.

Aneurysm rupture and growth are the conflicts of vascular protective factors and damage factors (20). The strong AWE is considered the sign of severe inflammation in the aneurysm wall. Existing studies revealed the correlation of AWE and atherosclerosis, and inflammatory cell invasion (7, 8, 21, 22). In this study, our findings from histological analysis complied with existing studies. The UIAs with AWE, compared with those without AWE, showed higher levels of MMP2 and CD68. In addition, the UIAs with AWE had the manifestation of a severe wall remodeling (Type II&III, or Type IV), with the general manifestations of inflammation infiltration, luminal thrombus and extremely thin hypocellular wall. The severe inflammation and wall remodeling suggested that the pathological characteristics of UIAs with AWE were similar to the ruptured aneurysms.

IL-1β, which is secreted after the cleavage and activation of GSDMD by caspase-1, significantly affects pyroptosis. As reported by existing studies, the IL-1β increased in atherosclerotic and infectious diseases (10–12, 23–26), in which the inflammation have essential significance. As opposed to the mentioned finding, the IL-1.ra can competitively combine the IL-1β receptor (27, 28), thereby reducing the biological effect of IL-1β, and it can act as an anti-inflammatory factor. According to this study, in patients with AWE UIAs had the up-regulated serum IL-1β and the down-regulated serum IL-1.ra. As revealed by the PPI analysis, GSDMD could interact with IL-1β and IL-1.ra. Moreover, high accuracy of IL-1β and IL-1.ra predictions was achieved for AWE. Notably, the levels of serum IL-1β and IL-1.ra were correlated with the relative levels of GSDMD, MMP2 and CD68 in aneurysm tissues. Impacted by the consistent comorbidities of the included patients, serum IL-1β/IL-1.ra and IL-1β/IL-1.ra in UIA tissues were found to have probably a consistent trend and could indicate the inflammatory condition of aneurysm wall (Figure 4D). According to the mentioned facts, pyroptosis may be critical to the process of UIAs. However, the *in vivo* and i*n vitro* studies should be conducted to confirm the assumption above.

In this study, the serum IL-1β and IL-1.ra were further integrated, and a novel biomarker, termed as serum IL-1.ra/beta ratio, was promoted. This biomarker could reflect the conflict of damage and repairment in the aneurysm wall. This study further demonstrated the predictive accuracy of IL-1.ra/beta ratio for AWE, and IL-1.ra/beta ratio was significantly correlated with the relative level of GSDMD in aneurysm tissues. Accordingly, IL-1.ra/beta ratio is considered to be a potential biomarker to predict the rupture or growth risk of UIAs, which should be explored in depth.

This study had several limitations. First, the source of serum cytokines might be variable. The regulated levels of serum IL-1β and IL-1.ra might be also attributed to other comorbidities. Although we took many other comorbidities into consideration, this problem would still limit our conclusion. Second, the resectable UIAs should exhibit a certain volume. In this current study, we only included UIAs >5 mm and included only 34 UIAs. The selection bias and the limited sample size might limit the conclusion of this study. Third, not all the aneurysms were entirely resected, which demonstrated that the pathological characteristics might not reflect the entire condition of UIAs. However, regardless of the mentioned limitations, this study suggested the correlation between AWE and pyroptosis in UIA tissues, and the pyroptosis-related factors might serve as potential biomarkers to predict the rupture or growth risk of UIAs.

## Conclusion

This study first indicated the correlation between AWE and pyroptosis in UIA tissues. The pyroptosis-related cytokines (IL-1β and IL-1.ra) were the serum cytokines correlated with the AWE. More pyroptosis-related proteins were identified in UIAs with AWE. The serum IL-1β, IL-1.ra and IL-1.ra/beta ratio was correlated with the relative levels of pyroptosis-related proteins and the inflammatory factors in UIA tissues. The factors correlated with pyroptosis (e.g., IL-1.ra/beta ratio) may serve as biomarkers correlated with aneurysm vulnerability.

## Data Availability Statement

The raw data supporting the conclusions of this article will be made available by the authors, without undue reservation.

## Ethics Statement

The studies involving human participants were reviewed and approved by Institutional Ethics Committees of Beijing Tiantan Hospital. The patients/participants provided their written informed consent to participate in this study.

## Author Contributions

QL and YZ: conception and design. WL, XM, JC, SM, LD, and NW: acquisition of data. QL, JW, PL, and SW: analysis and interpretation of data. QL: drafting the article. CZ and HH: critically revising the article. QL, YZ, CZ, WL, XM, JC, SM, LD, NW, JW, PL, HH, and SW: reviewing submitted version of manuscript. SW: approving the final version of the manuscript on behalf of all authors. HH and SW: study supervision. All authors contributed to the article and approved the submitted version.

## Funding

This study was supported by the National Natural Science Foundation of China (Nos. 81901197, 82071296, 81801158, 81671129, and 81471210), National Key Research and Development Program of the 14th Five-Year Plan (No. 2021YFC2500025), and Top Talent Support Program for young and middle-aged people of Wuxi Health Committee (No. 202014).

## Conflict of Interest

The authors declare that the research was conducted in the absence of any commercial or financial relationships that could be construed as a potential conflict of interest.

## Publisher's Note

All claims expressed in this article are solely those of the authors and do not necessarily represent those of their affiliated organizations, or those of the publisher, the editors and the reviewers. Any product that may be evaluated in this article, or claim that may be made by its manufacturer, is not guaranteed or endorsed by the publisher.

## Abbreviations

UIA: unruptured intracranial aneurysm
AWE: aneurysm wall enhancement
TIA: transient ischemic attack
MCA: middle cerebral artery
ICA: internal carotid artery
AcomA: anterior communicating artery
ACA: anterior cerebral artery
AR: aspect ratio
SR: size ratio
IL-1β: interleukin 1 β
IL-1. ra: interleukin 1 ra
TNF-α: tumor necrosis factor-α
GSDMD: gasderimin D
MMP2: matrix metalloproteinase-2.
