# Supplementary material for: Serum IL-1, Pyroptosis and Intracranial Aneurysm Wall Enhancement: Analysis Integrating Radiology, Serum Cytokines and Histology
Source: Front Cardiovasc Med. 2022 Jan 27;9:818789. doi: 10.3389/fcvm.2022.818789 (PMC8829005; doi:10.3389/fcvm.2022.818789)
Supplement: Supplementary file 1 [file Data_Sheet_1.PDF]

Supplementary materials to *Serum IL-1, pyroptosis and aneurysm wall enhancement: a study integrating serum cytokines and histological analysis*

Supplementary tables

Online Table 1. The function of cytokines in this study

No.	Cytokines	Function	No.	Cytokines	Function
1	CTACK	Other	24	IL-13	Other
2	Eotaxin	Other	25	IL-15	Other
3	Basic.FGF	Other	26	IL-16	Other
4	G-CSF	Other	27	IL-17	Proinflammatory
5	GM-CSF	Other	28	IL-18	Other
6	GRO-alpha	Other	29	IP-10	Other
7	HGF	Other	30	LIF	Other
8	IFN-a2	Proinflammatory	31	MCP-1	Other
9	IFN-gamma	Proinflammatory	32	M-CSF	Other
10	IL-1.alpha	Proinflammatory	33	MIF	Anti-inflammatory
11	IL-1.beta	Proinflammatory	34	MIG	Proinflammatory
12	IL-1.ra	Anti-inflammatory	35	MIP-1.alpha	Other
13	IL-2	Other	36	MIP-1.beta	Other
14	IL-2.Ralpha	Other	37	beta-NGF	Other
15	IL-3	Other	38	PDGF-BB	Other
16	IL-4	Other	39	RANTES	Other
17	IL-5	Proinflammatory	40	SCF	Other
18	IL-6	Other	41	SCGF-beta	Other
19	IL-7	Other	42	SDF-1.alpha	Other
20	IL-8	Other	43	TNF-alpha	Proinflammatory
21	IL-9	Proinflammatory	44	TNF-beta	Proinflammatory
22	IL-10	Anti-inflammatory	45	TRAIL	Proinflammatory
23	IL-12(P40)	Proinflammatory	46	VEGF	Other

Online Table 2. Inter-observer agreement analyses

Characteristics	Investigator 1	Investigator 2	Kappa or ICC (95% CI)
Irregular shape (Yes/No)	13/21	13/21	1.000 (1.000-1.000)
Aneurysm size	7.28 (6.26-9.47)	9.35 (7.10-10.70)	0.933 (0.037-0.985)
AR	1.44 (1.10-1.87)	1.26 (0.97-1.75)	0.949 (0.890-0.975)
SR	1.75 (1.30-2.34)	2.15 (1.47-2.74)	0.982 (0.329-0.996)

Abbreviations: ICC, intraclass correlation coefficient; CI, confidence interval

Online Table 3. The difference of cytokines† between UIAs with AWE and without AWE

Cytokines, *pg/mL*	With AWE	Without AWE	Fold change	*P* value
CTACK	1312.62 (465.62-1631.49)	978.64 (1162.55-1697.99)	1.41	0.327
Eotaxin	119.4 (29.165-121.95)	71.28 (69.54-178.42)	1.43	0.054
Basic.FGF	124.01 (64.58-119.91)	95.14 (102.54-143.29)	1.24	0.077
G-CSF	135.37 (66.63-212.79)	133.73 (119.54-177.2)	0.94	0.806
GM-CSF	7.82 (2.84-7.20)	5.37 (5.66-9.43)	1.23	0.103
GRO-alpha	449.66 (220.195-518.87)	378.82 (330.24-473.94)	1.01	0.506
HGF	747.32 (487.23-826.76)	685.23 (480.22-946.93)	0.56	0.766
IFN-a2	32.32 (15.77-33.42)	24.68 (28.68-40.12)	0.70	0.111
IFN-gamma	32.45 (13.67-29.82)	22.07 (24.03-37.69)	0.87	0.115
IL-1.alpha	70.16 (22.55-68.35)	46.84 (44.84-86.49)	1.27	0.195
IL-1.beta	24.81 (3.21-10.64)	7.21 (18.22-30.84)	3.04	<0.001§
IL-1.ra	1489.94 (2222.76-4021.50)	3057.3 (787.64-1706.64)	0.29	0.042§
IL-2	18.72 (6.09-19.74)	10.39 (11.49-25.15)	0.86	0.132

IL-2.Ralpha	154.54 (41.99-139.25)	93.42 (108.45-175.37)	1.32	0.039
IL-3	2.07 (1.13-2.55)	1.58 (1.63-2.51)	0.21	0.411
IL-4	4.91 (2.06-4.635)	3.11 (4.26-5.9)	2.19	0.007§
IL-5	18.49 (0-29.48)	2.75 (8.47-28.31)	0.10	0.219
IL-6	6.46 (3.03-10.22)	5.23 (3.74-9.81)	0.27	0.674
IL-7	20.41 (7.00-23.07)	14.08 (17.69-25.64)	1.34	0.063
IL-8	293.94 (41.80-334.48)	151.44 (179.53-453.43)	0.87	0.107
IL-9	114.91 (28.94-127.46)	84.45 (106.97-130.7)	0.29	0.146
IL-10	8.39 (1.79-10.66)	6.12 (6.87-9.9)	1.41	0.106
IL-12(P40)	381.73 (3.92-8.16)	262.45 (3.2-7.31)	0.68	0.156
IL-13	4.29 (134.85-363.77)	2.98 (255.79-454.65)	0.24	0.391
IL-15	543.61 (2.29-4.39)	448.2 (1.71-5.7)	0.15	0.431
IL-16	2201.97 (156.18-624.24)	1390.02 (357.44-685.02)	1.60	0.021†
IL-17	17.1 (605.80-1998.69)	12.32 (1427.23-2770.02)	1.40	0.104
IL-18	1240.28 (4.63-17.97)	711.33 (11.65-20.13)	1.83	0.08

IP-10	628.34 (475.57-1454.12)	320.22 (760.39-2597.15)	1.39	0.107
LIF	261.46 (91.02-684.41)	140.05 (339.19-1039.75)	1.05	0.046†
MCP-1	35.4 (50.84-254.31)	24.31 (146.73-318.19)	0.20	0.286
M-CSF	173.79 (13.63-48.02)	102.64 (26.69-59.4)	1.32	0.033†
MIF	20685.09 (1.19-4.74)	12385.63 (0-3.75)	1.77	0.115
MIG	212.62 (58.92-161.91)	140.74 (110.22-238.86)	1.17	0.421
MIP-1.alpha	5.89 (6846.31-24003.41)	4.68 (13635.59-25998.46)	1.21	0.506
MIP-1.beta	93.06 (95.29-331.31)	71.34 (89.58-374.98)	1.03	0.178
beta-NGF	2.62 (3.23-6.97)	1.85 (3.59-9.3)	0.42	0.123
PDGF-BB	2156.66 (35.93-110.89)	1913.76 (64.67-153.73)	0.85	0.552
RANTES	18568.24 (0.57-3.07)	18650.7 (2.33-3.49)	0.92	0.834
SCF	107.2 (1037.13-4670.59)	64.79 (1808.41-3191.85)	0.87	0.162
SCGF-beta	86391.9 (16019.56-23275.14)	70072.39 (16372.06-22546.17)	1.28	0.208
SDF-1.alpha	1119.2 (30.09-129.87)	836.29 (66.84-139.28)	0.91	0.278
TNF-beta	28.59 (26155.87-89317.71)	17.94 (61245.01-104849.22)	1.26	0.027†

TNF-alpha	158.82 (533.98-1309.22)	25 (888.04-1429.34)	9.65	<0.001[§]
TRAIL	41.1 (9.20-26.50)	29.42 (21.54-43.56)	1.90	0.012[┼]
VEGF	237.68 (11.37-47.59)	202.74 (77.46-176.35)	1.10	0.208
IL-1ra/beta ratio	0.47 (0.41-0.54)	0.19 (0.14-0.29)	2.47	<0.001[§]

†, The result was presented as the median and inter-quartile range.

┼, the cytokines were significantly different between UIAs with AWE and without AWE.

§, the cytokines with P < 0.05 and fold change > 2.

Abbreviation: AWE, aneurysm wall enhancement.

Online Table 4. The prediction of serum cytokines for AWE

	AUC	95% CI	*P* value
IL-1.beta	0.88	0.77-0.99	<0.001
IL-1.ra	0.90	0.80-1.00	<0.001
IL-4	0.78	0.62-0.94	0.007
IL-10	0.66	0.48-0.85	0.107
TNF-alpha	0.85	0.72-0.99	0.001
IL-ra/beta ratio	0.95	0.90-1.00	<0.001

Abbreviation: AWE, aneurysm wall enhancement; AUC, the aera under the curve; CI, confident interval.

Online Table 5. The correlation[†] of inflammatory factors in UIA tissues and cytokines

Cytokines in serum	Pyroptosis-related and inflammatory-related proteins in UIA tissues				
	IL-1.beta	IL-1.ra	MMP2	CD68	GSDMD
IL-1.beta	0.79┼	-0.18	0.58┼	0.58┼	0.63┼
IL-1.ra	-0.20	0.45┼	-0.40┼	-0.39┼	-0.42┼
IL-4	< 0.01	< 0.01	-0.04	-0.04	0.11
TNF-alpha	0.14	-0.13	0.11	0.11	0.27┼
IL-1ra/beta ratio	-0.67┼	0.62┼	0.44┼	0.54┼	0.72┼

†, the result was presented as the correlation coefficient.

┼, the correlation was significant.

Abbreviation: UIA, unruptured intracranial aneurysm.

Supplementary figures

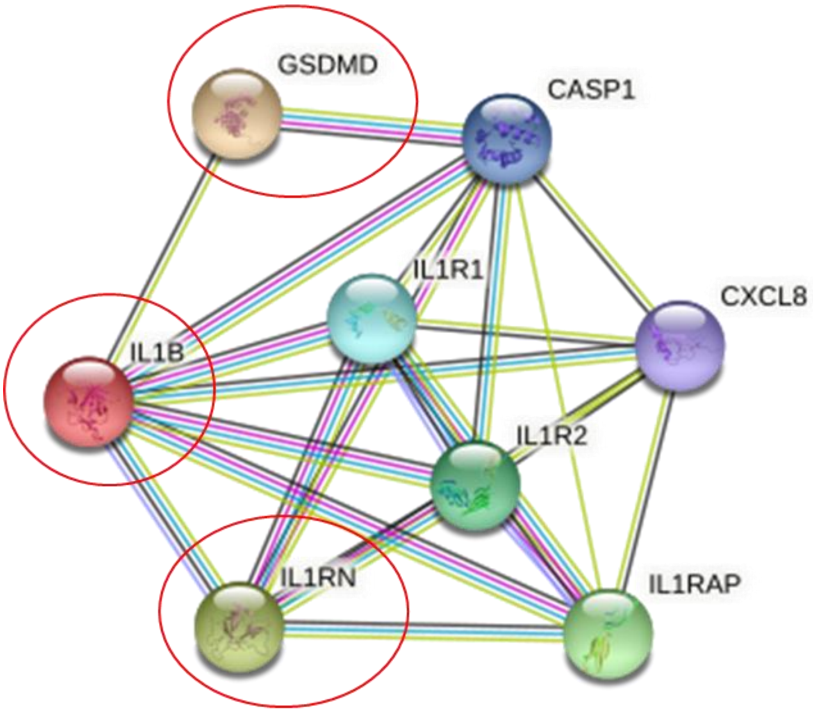

Online Figure 1. The interaction of targeted proteins. The protein-to-protein interaction analysis based on STRING database suggested that the GSDMD can interact with the IL-1β directly, and with the IL-1.ra indirectly. The red circles indicated the targeted proteins. GSDMD, gasdermin D; IL1B, IL-1β, interleukin 1β; IL1RN, IL-1.ra, interleukin 1 ra.
